# Do Peer Support Groups Facilitate Nutrition Information Practice and Nutritional Self‐Management in People Living With Type 2 Diabetes?

**DOI:** 10.1111/jhn.70158

**Published:** 2025-11-18

**Authors:** Jane McClinchy, Angela Dickinson, Wendy Wills

**Affiliations:** ^1^ School of Health, Medicine and Life Sciences University of Hertfordshire Hatfield UK

**Keywords:** embodied diabetes, Lay led peer support groups, nutrition information practice, practice theory, qualitative, solicited diaries

## Abstract

**Introduction:**

Nutrition information practice is fundamental to the process of nutritional self‐management for people living with Type 2 diabetes (PLWT2DM). Lay‐led, peer support groups have been proposed as a mechanism offering a source of nutrition information. However, there is limited research exploring how support groups facilitate nutrition information practices in PLWT2DM. The aim of this study was to explore the accessibility, acceptability and effectiveness of lay‐led peer support groups in the nutrition information practice of PLWT2DM.

**Methods:**

The study took a mixed methods qualitative approach through the lens of practice theory and information practice. Twenty participants (19 were PLWT2DM and one partner) were interviewed following a 4‐week diary data collection period where peer support interactions relating to nutrition information practices were recorded by participants. Data were analysed using thematic analysis.

**Results:**

Participants used their experiences of advice from a dietitian and structured education sessions to emphasise the relative influence of peer support groups on their nutrition information practices. Particularly their accessibility, ongoing nature and facilitatory environment enabling social interactions. However, key competencies participants needed were feeling that diabetes was an embodied (integrated) part of everyday life, group communication skills and facilitation skills (considered essential for group leaders).

**Conclusion:**

Peer support groups provide a useful addition to the nutrition information landscape of PLWT2DM. However, the stage of disease embodiment (integration) and skills in social interaction impacted on their perceived effectiveness. Dietitians need to consider the benefit and potential longevity of peer support for PLWT2DM particularly since the COVID‐19 pandemic, as a shift towards online formats for peer support groups have changed their availability and possible effectiveness.

## Summary

- The accessibility, ongoing nature, and opportunities to share ideas in peer support groups help people living with Type 2 diabetes to undertake nutrition information practice to self‐manage their condition.
- Peer support groups should form part of the information landscape proposed by dietitians and other healthcare professionals working with people living with Type 2 diabetes.
- Skills and dispositions for social interactions will influence when, whether and how people living with Type 2 diabetes join a peer support group.

## 1 Introduction

People living with Type 2 diabetes (PLWT2DM) need to make multiple daily decisions about what to eat to self‐manage their condition. Being able to undertake self‐managed practice is crucial in being able to make these decisions [1] and information practice is a core element in the process of self‐management [2].

While information itself does not lead directly to behaviour change [3] information is needed to be able to undertake self‐managed practice as it is a key precursor in behaviour change models [4]. Although PLWT2DM should receive nutritional information and advice from a dietitian on diagnosis and throughout their treatment [5] current limitations in service provision make this unachievable [6]. In the United Kingdom, NICE [7] recommend that PLWT2DM are offered structured education on diagnosis. However, in the 2023–24 National Diabetes Audit NHS Digital [8] only 8.6% of newly diagnosed patients with T2DM who were offered the opportunity to attend, did so. Research suggests that perceived needs for structured education and practical barriers impacting on attendance have been found to be the main reasons for non‐attendance [9, 10]. Therefore, PLWT2DM need to undertake their own nutrition information practice to support their nutritional self‐management.

Information gathering is a basic human need [11, p. 1], an everyday and instinctive practice [12], which is influenced by social and cultural factors [11, 13]. Lloyd et al. [2] explain that people living with long term conditions use health information practices ‘to inform their decision making in relation to the day‐to‐day management of their chronic illness′. They make use of an ‘information landscape′ which incorporates a wide range of activities, sources and skills to self‐manage [2, pp. 7, 1]. Nutrition information practices have been found to be entangled with food and other everyday household practices [14]. Wills et al. [14] proposed that rather than each food practice being distinct and discrete, they were ‘entangled′ with other non‐food practices. For example, when undertaking food hygiene practices in domestic kitchens, checking food labels or identifying foods which need disposal were undertaken along with other practices unrelated to food provisioning such as helping children with homework or feeding pets. That is that each practice was hidden, tacit and undertaken seamlessly [14, p. vii].

Peer support has been defined as *‘…the provision of emotional, appraisal, and informational assistance by a created social network member who possesses experiential knowledge of a specific behaviour or stressor and similar characteristics as the target population…′* [15, p. 329]. Peer support has been found to promote self‐management skills [15] and improve clinical outcomes [16]. While peer support activities for PLWT2DM may be incorporated into formal structured education sessions [17, 18], lay led peer support is normally considered an informal source of information for PLWT2DM [17, 19, 20] that is available outside of the normal healthcare sources of information [18]. Such support, therefore, may be a more acceptable and accessible source of information for patients [21, 22]. Heisler [19] hypothesised that peer support would provide informational and emotional support and mutual reciprocity. This would impact attitudes towards diabetes self‐care which would improve self‐care, glycemic control and decrease the use of healthcare services. The strengths of lay led peer support approaches are their flexible, informal nature, being non‐hierarchical [23] and provision outside the formal medical model of healthcare [24]. With the concerns about attendance at formal structured education sessions [8, 9, 10] and limited dietetic resources [6], lay led peer support groups could provide a relevant alternative that PLWT2DM may find more acceptable [25], however, there is limited research in this area exploring the experiences of PLWT2DM.

The aim of this study was to explore the accessibility, acceptability and effectiveness of lay led peer support groups as an informal source of nutrition information for PLWT2DM.

## 2 Methods

The study took a mixed methods qualitative approach with practice theory and information practice providing the underpinning theoretical framework for the study [2, 26, 27].

Practice theory is an approach increasingly used by researchers to help explain tacit and hidden everyday behaviours that are generally taken for granted [28]. Practices are made up of three main interconnected elements: materials, meanings and competencies [26, 27]. Changing the way an individual experiences these components may impact on the way they act out their everyday practices [26, p. 12). That is materials, meanings or competencies from one practice may impact another practice and practices themselves may impact on other practices. For example, Shove et al. [27, p. 93] refer to the practice of incorporating frozen foods in day‐to‐day food provisioning and how their use impacted on other practices such as ‘cooking, parenting and managing the home′. Similarly, they propose that the practice of weight monitoring as part of individuals′ health management impact on food provisioning and activity practices (ibid p. 110). While Lloyd et al. [2] who explored health information practice in people living with long term health conditions identified health literacy as a key competency in being able to undertake health information practice, with the level of health literacy possessed by an individual impacting on their ability to undertake information practice.

Although practice theory has been applied to food practices [14, 26, p. 93, 29, p. 75, 30, 31] and health information practices [2], there is limited research exploring nutrition information practices in PLWT2DM. In this current study rather than the theoretical framework of practice theory being pre‐determined, the components of practices relating to dietary management were revealed through the process of thematic analysis.

PLWT2DM and or their spouse were recruited from support group venues (*n* = 15), via support group communications (*n* = 4) or the participants′ workplace (*n* = 1). The chairs/facilitators of potential support groups identified through Diabetes UK and through professional networks were contacted and offered a nutrition talk. A total of 75% of participants were recruited this way. All were asked to record interactions with nutrition information over a 4‐week period in an unstructured diary specifically developed for this study. Participants were given a diary pack including an A5 notebook with lined pages, pencils, coloured pens and glue stick and were supported to make relevant diary entries through twice weekly email reminders. After the diary period, the diaries were collected and reviewed by the lead author. This review was used to individualise an interview topic guide to ensure that any aspects in the diary requiring clarity were discussed alongside a core set of interview questions. The interview began with the participant being asked to ‘*talk me through your diary*'. These hour‐long interviews were conducted in participants' own homes or a convenient location of a participant's choice. Diaries were scanned into pdfs and interviews were audio recorded. Both had identifying information redacted and were transcribed verbatim.

Data were uploaded into NVIVO and analysed using the process of thematic analysis as described by Braun and Clarke [32]. A key feature was familiarisation using an iterative process of reading and coding textual data within NVIVO. Hard copies of the diaries were also annotated with main topics colour coded. As analysis progressed the codes were then grouped together into conceptual themes. These were further developed by exploring how they related to each other. Discussion of theme and conceptual development and how they related to the developing conceptual framework occurred throughout the analytic phase of the study with all co‐authors, with lack of consensus being resolved through discussion.

Informed consent was recorded at the start of the study and ethics approval was given by the University of Hertfordshire Health and Human Sciences Ethics Committee, protocol number aLMS/PG/UH/00099(1). Further details of the method are described in McClinchy et al. [33].

## 3 Findings

### 3.1 Participants

Twenty participants (19 were PLWT2DM and one partner) were recruited through lay led peer support groups identified on the Diabetes UK website (Diabetes UK Local Support Groups: https://www.diabetes.org.uk/support-for-you/community-support-and-forums/local-support-groups), through personal contacts from across the East of England, and from the researcher's workplace. Characteristics of participants are summarised in Table 1. Age range was from 52 to 84 years and time since diagnosis from 6 months to 23 years. Data consisted of 19 diaries and interviews. One couple both living with T2DM took part jointly. All names are pseudonyms.

**Table 1:** Participants.

Pseudonym	Age range in years at interview	Time since diagnosis in years	Personal relationships	Recruitment venue	Lay led Peer support group attender/leader (group number)
Andrew	70–74	23		Support Group	Leader Group 1
Christopher	65–69	7	Married to Danielle	Support Group email	Previous leader
Danielle	65–69	N/A^a^	Married to Christopher	Support Group email	Experienced peer support in structured education
Edward	75–79	5		Support Group email	Attender Group 2
Frances	55–59	0.5		Researcher workplace	Experienced peer support in structured education
Gary	60–64	7		Support Group	Leader Group 3 with Isobel
Helen	70–74	2		Support Group	Attender Group 3
Isobel	55–59	21		Support Group	Leader Group 3 with Gary
Jennifer	65–69	0.5		Support Group	Attender Group 3
Lisa	50–54	1		Support Group	Attender Group 2
Matthew	65–69	5	Married to Naomi^b^	Support Group	Leader Group 2
Naomi	70–75	11	Married to Matthew^b^	Support Group	Attender Group 2
Oscar	55–59	1.5		Support Group	Leader Group 5 with Penelope
Penelope	70–74	15		Support Group	Leader Group 5 with Oscar
Ruth	55–59	1		Support Group	Attender Group 6
Susan	60–64	12		Support Group	Attender Group 1
Theresa	50–54	1		Support Group	Attender Group 7
Victoria	50–54	7		Support Group	Attender Group 7
William	80–84	20		Support Group	Leader Group 8
Yvonne	76–79	10		Support Group	Attender Group 9
Average	65	8			

^a^ Danielle did not have diabetes.

^b^ Mathew and Naomi completed a joint diary and chose to be interviewed together.

Most participants experienced informal face‐to‐face lay led peer support held in places such as in non‐healthcare community settings for example a community room provided by a supermarket. One peer support group was held in a room in a local community hospital. However, Danielle and Frances had experience of peer support within the structured education sessions they attended. Eight participants had experience of leadership either in the form of chairing a peer support group meeting (Andrew and Matthew) or as a facilitator (Christopher, Gary, Isobel, Oscar, Penelope, William).

Peer support groups were identified as key sources of nutrition information supporting their nutritional self‐management. For example, Gary said:

‘I think diet would be the top subject that people want to talk about [in the group sessions].'

Data are discussed below using the three main elements of practice theory: materials, meanings and competencies as the main theme headings.

### 3.2 Materials

This section explores the nutrition information practice themes relating to materials. The materials identified by participants were those relating to knowledge or information imparted by healthcare professionals such as dietitians and structured education facilitators and through social interaction in peer groups. Indeed, the main topic of conversation in the peer support groups was nutritional management.

Participants used their experiences of the limited availability of advice from a dietitian and the finite nature of structured education sessions to emphasise the importance of the accessibility and ongoing nature of peer support groups.

Although some participants had seen a dietitian this service was not available to everyone. For example, when Jennifer asked for guidance on what to eat from her GP she was told *'we don't do that anymore'*. Despite structured education being part of the agreed treatment for PLWT2DM, some participants had not been offered the opportunity to attend sessions. For example, Gary said:

‘…whether it's something they forgot to mention it to me …or whether its financial pressures…but there are some of us who've been offered it and some who haven't.'

**Figure 1:**
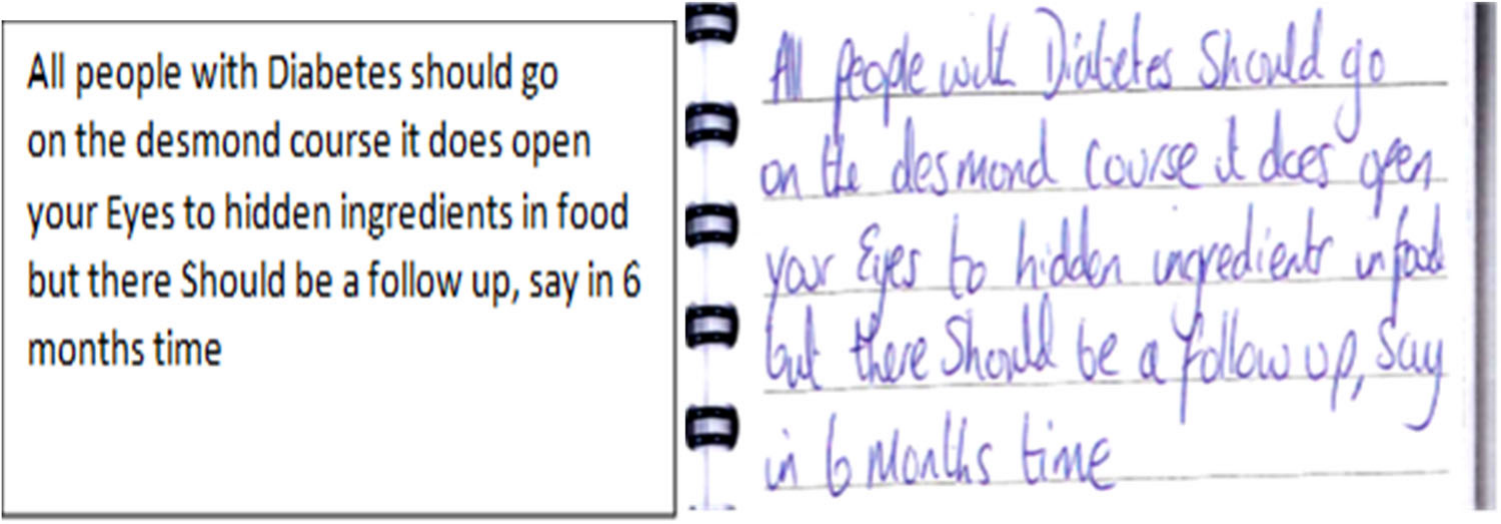
Teresa Diary referring to her experiences of the finite nature of structured education. Facsimile left, transcript right.

Participants referred to the limitations of the finite availability of nutritional advice from healthcare professionals and the structured education courses they had been on. They likened the feeling to being let down and abandoned by services that should be providing support. For example, Naomi said once someone with T2DM has had advice from a dietitian or attended structured education ‘[you are] *left to kind of fend for yourself'*. Theresa wrote in her diary that while she valued the information she learned in DESMOND (a structured education course), she felt that there should be a follow up afterwards: Figure 1.

During the interview Theresa was more vehement in expressing her feelings about the impact of the structured education course:

‘…you go on the course, and then you're just dropped from a big height, which is really sad because they teach you lots of things and make you look at things [on the structured education course], but then after that you are on your own, and it's quite sad.'

However, participants also identified that even when support group meetings are regular, this does not suit all as not everyone can or wants to attend regular support meetings. Matthew (who is chair of his local support group) explained that about 10% of his membership attend the meetings, the rest keep in touch with his monthly newsletter. He said some ‘…*live too far away, or don't have the transport, or don't have somebody who can bring them or who don't want to come, some people don't want to come to meetings that's not their thing'*.

### 3.3 Meanings

The nutrition information practice theme relating to meanings focussed on social factors experienced in the peer group settings, identifying that the facilitatory environment enabled social interactions.

Being able to share ideas and experiences was important. Jennifer wrote in her diary after her first peer support group meeting that she valued *‘meeting other like‐minded [people] and listening to their stories'*. Victoria said that *‘a lot of people have got other ideas…'* and Helen explained that it was not just the facilitators or invited speakers but that it was the other group members that ‘*give input'* into the discussions. The main topic of conversation was food; people talked to each other about how they were managing, and from this they were able to *‘pick up suggestions'* (Oscar). For example, Ruth recalled suggesting mashing carrots up with potatoes as a way of increasing vegetable intake. She said:

‘…and you are just going, “Well, did you not think of maybe putting some carrots in there?” “I don't like carrots.” “Yeah, but if you mash them up, and mix them up with the potatoes…” You know, just giving each other ideas.'

(Ruth)

Ruth went on to explain that the normal power relationship that exists when you have a healthcare professional who is trying to motivate you is replaced by a feeling of trust, support and friendship between group participants:

But by going to the group … You can talk to each other … Because you're not being shouted at by somebody to lose weight, to do this, to eat that, you're talking between each other. You're like just friends.

(Ruth)

**Figure 2:**
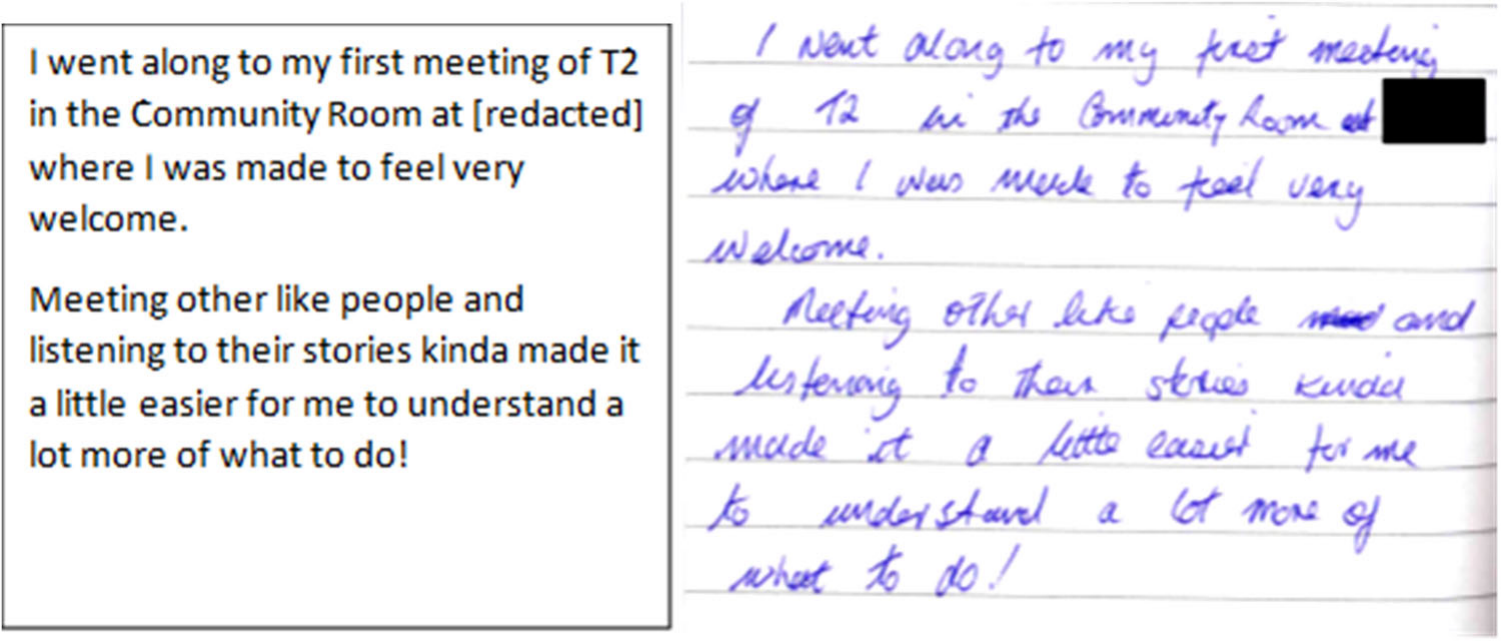
Jennifer describing her first peer support group experience. Left Diary facsimile, right transcription.

Jennifer who had *‘blanked'* out her diabetes and reported being in denial recorded in her diary her experience of first attendance at her local peer support group (see Figure 2).

Conversely not everyone valued the social interaction with peers in a group setting. Yvonne described how as she did not have anyone to attend the meeting with, she did not persist with attending a peer support group as she *‘didn't really get anything out of it'*. Frances found the group setting challenging as she found that while she was engaging in the group activities other members did not appear to be *‘interested in it'*.

### 3.4 Competencies

This nutrition information practice theme referred to competencies that were needed to undertake information practice activities relating to peer support. For example, a key competency needed to be able to engage with peer support was having an acceptance of their T2DM which enabled them to integrate their diabetes into their daily lives (referred to as embodied diabetes). Other skills identified were, being able to communicate with others in a group setting and being able to support and facilitate groups, considered essential for group leaders (*n* = 7 current group leaders and *n* = 1 previous group leader).

### 3.5 Embodied Diabetes

The stage at which participants were in their trajectory of incorporating their diabetes influenced their ability to engage in information practice. Those with more recent diagnoses described feeling *‘like someone had hit [them] over the head'* (Theresa interview) they ‘*blanked it out'* (Jennifer diary) and were *‘in denial'* (Jennifer interview). They were not able to engage in nutrition information practice. However, once they had come to terms with the diagnosis, they were better able to engage with and attend a diabetes support group.

Similarly, Matthew and William who were both support group facilitators noticed how people reacted to a diagnosis or potential diagnosis with fear and anger. For example, William noticed at a community diabetes testing event that following a random blood glucose result of 17 mmol/L a participant ‘*was in tears'*. Matthew went on to note how these feelings can prevent engagement with diabetes information until they feel they are able to take on advice and guidance. This particularly impacts on referrals to diabetes structured education sessions and the consequent scheduling of these. He said:

‘the problem I have is because they will only take them when they're newly diagnosed, somebody who may be in denial or may be in shock who doesn't want to go to the course when they're newly diagnosed, in six months' time they may be, that's, they're mentally receptive to that type of stuff, that's when they need to be going, and it's not offered any more. And the scandalous thing is they cancel [structured education] courses because they can't get enough people. Because a lot of the newly diagnosed people don't take it up.'

### 3.6 Conversations

Having the skills and desire for social interactions facilitated engagement in peer support groups settings. Jennifer talked about the importance of listening skills which William went on to explain was crucial in a group setting *‘so that [everyone] can hear what that person is saying…as it might be important to [them]'*. He also explained how talking would lead to a *‘conversation'* (William). For example, Helen talked about having conversations saying, *‘somebody might throw something in and we would have a conversation…I've learned quite a lot from different people'*.

Not all participants found the group provided social interaction. Yvonne was intending to go to the group with a friend, however, as her friend was no longer able to attend Yvonne went on her own. She did not find the group experience enjoyable or useful. She said:

‘…I don't know what it was. Well, you talked, but I went twice, and [the group leader] went round [the group and asked each of us], ‘Now tell me about your diabetes, and how many tablets do you take, how often do you inject,’ and it was so boring. Then she gave us a sheet at the end, but we could have had just a sheet, we didn't [learn anything new]'

### 3.7 Facilitation Skills

A key aspect of the success of the support group was the skill of the facilitators in making the sessions engaging and interesting. Isobel, Gary, William, Penelope and Oscar were facilitators of their support groups and Andrew and Matthew were chairs of a support group meeting. Discussions with participants emphasised the coordination skills the facilitators possessed. However, whilst these skills were considered essential the possession of these did not appear to have been considered in the nomination process as group leaders. Instead, the process was based on an experience of living with Type 2 diabetes, a willingness to take on the role and a self‐perception of being able to lead the group. For example: For William, the selection process undertaken by a diabetes nurse at his GP practice was based simply on having lived with diabetes; Isobel identified her own disposition of being a leader and her interest in supporting the management of diabetes that led her to proactively offer to take part, while Matthew ended up taking on the role by default.

### 3.8 William Said

‘I got a bit of arm twisting from the diabetic nurse. She said, ‘You've been diabetic a few years, I think you might be interested in this.’ I looked at it, and she sort of said, ‘You'd be an ideal candidate for it.' So I went along to a meeting, in [the next town], and there were a few of us there, and that was a two‐day course'

Isobel said:

Well, I have a policy, I have a policy for most things, and uh, so my policy is to volunteer for any [activity] that will help people with diabetes as long as nobody makes me swallow a pill.

Matthew said:

‘I was diagnosed; Naomi had already been going to the group so I thought I would have to go. I turned up for my first meeting to be told that the chairman had died, and they had to elect a new chairman by the next meeting. The next meeting nobody was prepared to put themselves forward, so I did. I was chairman though I knew nothing about diabetes.’

Participants did not articulate the skills required to become a group facilitator beyond living with Type 2 Diabetes and being willing to take on the role. However, those who were leaders identified a range of skills that they utilised when running the group. William and Matthew both perceived their roles as providing a setting for facilitating discussion and raising awareness. Specifically, William was concerned that he would be expected to provide further guidance and advice on diabetes. He said:

'I don't want to be in the situation where they expect me to be the know‐all, know everything about it. It's just to make them aware of some things, and get a little bit of feedback, and have some literature to pass on.’

Matthew felt that it was important for leaders to have some knowledge about Type 2 Diabetes saying:'

*‘I don't think it was necessarily good for the group that I didn't know anything.'*

However, Isobel took her perception of her role a step further. She felt that she needed to be able to provide some advice and guidance, so she undertook her own research into the latest advice for Type 2 Diabetes. She said:

‘And if I'm going to be a role model there's no point me just walking about… going this is what I do it's all right for me chaps, I actually need to be well educated. So uh, I'd already done quite a bit of research because I am a researcher at heart, so I built on that, extended that, read some more books that were out…’

While William, Isobel and Matthew took pride in their roles as leaders of peer support groups and appeared to enjoy the opportunity, not all who had taken on the role of peer support group leader enjoyed the role or found it was in their skill set. For example, Christopher said *‘it wasn't really my forte engaging other people I didn't enjoy that at all'*.

## 4 Discussion

Use of the practice theory framework helped to reveal the specific nature of peer support groups and why they were a key aspect in participants' nutrition information practice. The three elements of practice theory revealed different components important to ensure the accessibility of peer support groups and their value to participants. The group setting was an important component of the participants' nutrition information landscape. The format also filled the nutrition information void for PLWT2DM due to lack of a nutrition information service in primary care and limited referral to a structured education programme. Peer‐led sessions had the additional benefit of providing opportunities for social engagement for participants.

Participants found that the accessibility, ongoing nature, and opportunities to share ideas in the peer support groups helped them to live with their diabetes and with their nutritional self‐management. This contrasted with the finite nature of structured education sessions where there were limited opportunities to share ideas and to compare experiences over time. However, while there was an emphasis on the importance of getting to know group members and feeling comfortable with sharing experiences, this required a preference and willingness to share and skills in being able to communicate in a group. That is, skills and dispositions for social interactions influenced when, whether and how PLWT2DM joined and attended a peer support group. While the hidden nature of everyday food practices [14] meant that participants did not often refer explicitly to their nutritional self‐management.

The study highlighted the lack of availability of nutrition information provided by healthcare professionals such as dietitians for some participants [6], the limitations of the structured education referral process in the United Kingdom [9, 10]. and the inherent finite format of the structured education provision [34]. The peer support groups in the current study were all held face‐to‐face mainly in community settings. The data collection for the study was undertaken before the COVID‐19 pandemic. Since the COVID pandemic only one Diabetes UK support group has returned to being face‐to‐face while other groups are available in an online format [35]. While peer support can be in the format of regular online meetings as in the Diabetes UK support groups or as online forums [36] the need for technological skills and moderators could limit social engagement [37]. However, there is research suggesting that synchronous lay led online group meetings for PLWT2DM could be an acceptable format [38].

The participants valued support groups that played an important role in supporting their nutrition information practice by providing social interaction, sharing stories and having conversations as identified by Heisler [19]. However, not all participants valued or wished to take part in a group conversation. While there is limited research exploring preferences for and confidence in group communication in peer support groups, there is research which has explored this aspect in structured education sessions suggesting this may influence attendance. For example, Horigan et al. [9, p. 22] identified that a reason for not attending structured education may be ‘feeling uncomfortable about the idea of joining a group'. A key success of peer support in nutrition information practice identified in the study was the sharing of ideas and problem solving which without the presence of healthcare professionals may have led to a feeling of empowerment [39] and a greater confidence in the ability to nutritionally self‐manage.

While the conversational and listening skills of facilitators were key in supporting PLWT2DM to engage in the group format, the stage of acceptance, embodiment and integration of diabetes impacted on the ability to interact as much as the desire for and ability to benefit from nutritional information and engage in social interaction. Those who were more recently diagnosed had identified that it had taken them some time to come to terms with their diabetes and to engage with the peer support. Therefore, a level of embodiment of Type 2 diabetes is needed for engagement in activities to support self‐care. Other research has also identified that to undertake self‐management, there is need for acceptance, embodiment and integration of the long‐term condition [40, 41, 42, 43]. For example, St. Jean [42] who utilised a diary approach in her study exploring health information with [35]. 34 PLWT2DM, identified the impact of time on the awareness of the need for information. While the study did not focus specifically on nutrition information, in common with the current study, they identified a change in awareness of and need for information over time following a diagnosis of Type 2 diabetes [42].

Several of the participants were facilitators of their support group, a role which although they took on opportunistically was one which they were proud to undertake, identifying skills or activities which they used to ensure that the groups ran effectively. While group attendees were positive about their group facilitators, research suggests that there are challenges in ensuring lay‐led groups enable and support all participants [23] and that the skills and personality types of facilitators are important in ensuring a culture of equality and ongoing success of the group [44].

**Figure 3:**
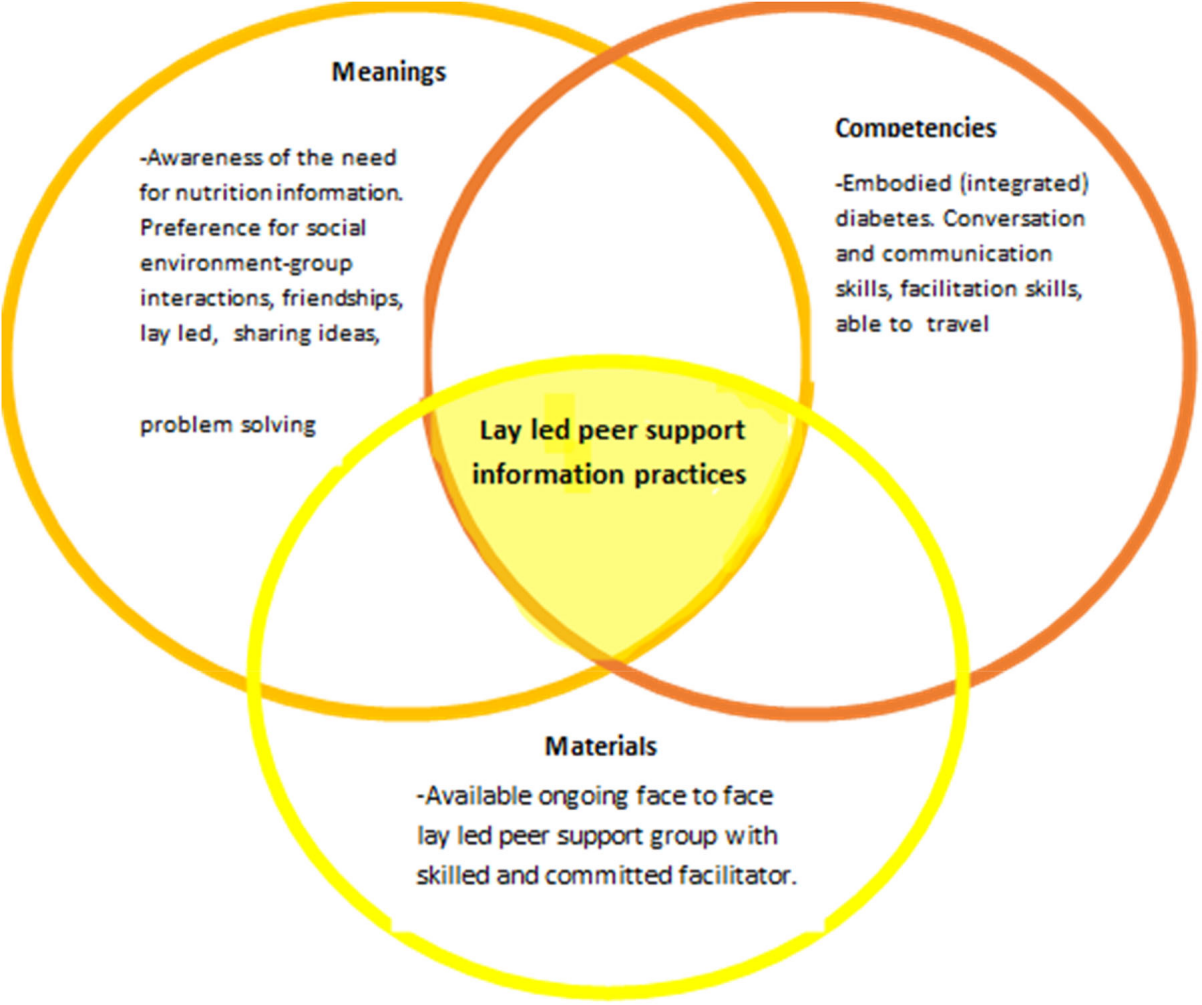
The components of lay led peer support information practices experienced by PLWT2DM (adapted from Shove et al. [27]).

The current study identified practice theory typologies for peer support information practice. The value of the social aspect, the need for skills in facilitation (for group leaders) and conversation and a degree of embodied diabetes to be able to engage with peer support groups as part of nutrition information practices are emphasised. These are collated together and illustrated in Figure 3 below.

### 4.1 Limitations

Although the study benefitted from the approach to recruitment focusing on the support group setting in being able to undertake an in‐depth exploration of peer support groups, a wider approach may have identified different themes and strengthened disconfirming instances in preferences for the peer support group format. Also, the approach may have resulted in the recruitment of those who were highly engaged in the self‐management of their T2DM and preferred the use of peer support groups to support their information practices.

## 5 Conclusion and Recommendations for Practice

Using practice theory coupled with the concept of integration and embodiment of a long‐term condition helped to reveal nutrition information practices amongst the participants. The components of practice theory were identified in the use of peer support groups as part of the participants' nutrition information practice. Particularly emphasised were the availability of nutrition information through ongoing lay led groups; the meaning for taking part as being the social interaction and sharing ideas; and the competencies as being conversation skills, degree of embodied (integration) of diabetes and facilitation skills for those who were group leaders. Peer support groups provide a useful addition to the nutrition information landscape of PLWT2DM and should be part of the menu of nutrition information sources recommended to PLWT2DM by dietitians. However, dietitians should take into account the stage of embodiment (integration) of diabetes and the desire for and skills in social interaction when proposing group education sessions and peer support groups. For those setting up groups their facilitatory skills are key in the ongoing success of a peer support group. Although post the COVID‐19 pandemic many peer support groups that were previously available face to face are now available online we do not know the accessibility and acceptability of such groups to PLWT2DM. Further research is needed exploring the longevity of peer support groups and the new online format for the majority of groups.

## Author Contributions

All authors contributed to the manuscript substantially and have agreed to the final submitted version. Jane McClinchy collected the data as part of her PhD. Angela Dickinson and Wendy Wills provided supervisory input. All authors contributed to the concept of the article, the analysis and data interpretation.

## Ethics Statement

Ethics approval was obtained from the University of Hertfordshire Health and Human Sciences Ethics Committee, protocol number aLMS/PG/UH/00099(1).

## Conflicts of Interest

Jane McClinchy and Angela Dickinson did not receive any financial support for this study. Wendy Wills University of Hertfordshire is supported by the National Institute for Health and Care Research (NIHR) Applied Research Collaboration East of England (NIHR ARC EoE) at Cambridgeshire and Peterborough NHS Foundation Trust. The views expressed are those of the authors and not necessarily those of the NIHR or the Department of Health and Social Care.

## Data Availability

The data that support the findings of this study are available on request from the corresponding author. The data are not publicly available due to privacy or ethical restrictions.
